# The Risk of Cancer among Taiwanese Female Registered Nurses: A Nationwide Retrospective Study

**DOI:** 10.1371/journal.pone.0068420

**Published:** 2013-07-16

**Authors:** Cheng-Che Shen, Yu-Wen Hu, Li-Yu Hu, Chin-Lin Perng, Tung-Ping Su, Chung-Jen Teng, Sang-Hue Yen, Cheng-Hwai Tzeng, Tzeon-Jye Chiou, Chiu-Mei Yeh, Tzeng-Ji Chen, Wei-Shu Wang, Pan-Ming Chen, Chia-Jen Liu

**Affiliations:** 1 Department of Psychiatry, Chiayi Branch, Taichung Veterans General Hospital, Chiayi, Taiwan; 2 School of Medicine, National Yang-Ming University, Taipei, Taiwan; 3 Cancer Center, Taipei Veterans General Hospital, Taipei, Taiwan; 4 Institute of Public Health, National Yang-Ming University, Taipei, Taiwan; 5 Department of psychiatry, Yuli Veterans Hospital, Hualian, Taiwan; 6 Division of Gastroenterology, Department of Medicine, Taipei Veterans General Hospital; 7 Department of Psychiatry, Taipei Veterans General Hospital, Taipei, Taiwan; 8 Division of Oncology and Hematology, Department of Medicine, Far Eastern Memorial Hospital, Taipei, Taiwan; 9 Department of Biomedical Imaging and Radiological Sciences, National Yang-Ming University, Taipei, Taiwan; 10 Division of Hematology and Oncology, Department of Medicine, Taipei Veterans General Hospital, Taipei, Taiwan; 11 Department of Family Medicine, Taipei Veterans General Hospital, Taipei, Taiwan; 12 Department of Internal Medicine, National Yang-Ming University Hospital, Yilan, Taiwan; 13 Department of Psychiatry, Su-Ao Branch, Taipei Veterans General Hospital, Taipei, Taiwan; CUNY, United States of America

## Abstract

**Background:**

To evaluate the risk of cancer among Taiwanese female registered nurses (RNs) using a nationwide population-based dataset.

**Methods:**

We recruited female RNs without antecedent cancer from the Taiwan National Health Insurance Research database during 2000–2010. Standardized incidence ratios (SIRs) of cancer were calculated. We also compared rates of Papanicolaou (Pap) smear use between the RNs and the general population matched by age and sex.

**Results:**

A total of 2,077 cancers developed among 184,809 female RNs, with a follow-up of 1,371,910 person-years (median follow-up of 7.86 years), leading to an increased SIR of 1.10 [95% confidence interval (CI) 1.05–1.15]. RNs aged between 40–59 years also had a significantly increased SIR (1.14, 95% CI 1.08–1.21). For specific cancer types, RNs had an increased SIR for breast (1.28, 95% CI 1.19–1.37), thyroid (1.26, 95% CI 1.10–1.43), lung and mediastinum (1.36, 95% CI 1.13–1.62), and uterine cancers (1.23, 95% CI 1.01–1.49). A decreased SIR was found for cervix (0.48, 95% CI 0.37–0.61) and liver and biliary tract cancers (0.68, 95% CI 0.50–0.90). Pap smear use averaged 5.80 times per person among female RNs aged 35 years or older and 5.50 times per person in the age-matched control group (*p* = 0.009).

**Conclusion:**

This study found that overall cancer risk was higher among female RNs than general population. For individual cancers, the risks of breast, lung, thyroid and uterine cancer were higher and the risks of cervix and liver cancer were lower than general population. The lower risk of cervical cancer might be partially explained by the increased use of Pap smears in the RNs group. Further large, unbiased population-based prospective studies are needed to investigate the association between nurses and cancer risk and identify the risk factors of cancer in nurses.

## Introduction

Nursing is a profession within the health care system focused on the care of individuals, families and communities so they may attain, maintain, or recover optimal health and quality of life. It is also one of the oldest female occupations in modern society, providing work for a large number of women. Being a nurse implies the possibility of exposure to over 100 agents in their work environment that are associated with increased risks of cancer, including biological, chemical, physical, and psychological health hazards. [1] Furthermore, the work performed by nurses also makes shift and night work inevitable. Shift and night work has been associated with an increased risk of chronic nonmalignant diseases, such as gastrointestinal disorders, cardiovascular disease, and diabetes. [2], [3] Besides, exposure to light at night and a disruption of the circadian rhythm have been hypothesized to affect cancer risk. [4]–[10] On the other hand, being a nurse also has defined aspects of lifestyle, with a possible influence on cancer risk.

The aim of the current study was to determine whether female registered nurses (RNs) in Asia are associated with a higher risk of cancer than general population. This study was a population-based retrospective cohort study using a database derived from the National Health Insurance (NHI) system in Taiwan.

## Materials and Methods

### Ethics Statement

The Institutional Review Board of Taipei Veterans General Hospital approved this study (2012-12-002AC). Written consent from study objects was not obtained, as the NHI dataset consists of de-identified secondary data for research purposes and the Institutional Review Board of Taipei Veterans General Hospital issued a formal written waiver for the need for consent.

### Data Sources

The NHI program, which began in 1995, is a mandatory universal health insurance program, offering comprehensive medical care coverage to all Taiwanese residents with a coverage rate of up to 98%. [11], [12] The program includes coverage for outpatient, inpatient, emergency, and traditional Chinese medicine services, as well as prescription drugs. This study used the NHI research database, which is managed and made available to the public by the National Health Research Institute (NHRI) of Taiwan. The NHI database of catastrophic illness includes comprehensive enrollment information for all patients with severe diseases, such as cancer, who have received copayment exemption under the NHI program. Confidentiality is maintained according to the data regulations of the Bureau of National Health Insurance and the NHRI.

### Study Population

We conducted a retrospective cohort study from January 1, 1996 to December 31, 2010. Female RNs were selected from the Registry for medical personnel (PER) and the Longitudinal Health Insurance Database 2000 (LHID 2000) from January 1, 2000 to December 31, 2010. The PER contains basic data of all medical staff in Taiwan, such as birthday and sex. The LHID 2000, which contains all original claims data for 1,000,000 subjects, is a representative database randomly selected from the 2000 Registry of Beneficiaries under the NHI program. This study recruited female RNs aged 20 years or older who had no prior malignancies from January 1, 1996 to December 31, 1999.

### Statistical Analyses

The main dependent variable was occurrence of cancer, as reported in the Registry for Catastrophic Illness. For a diagnosis of cancer to be reported in the Registry, histological confirmation is required. Cancer was defined according to the International Classification of Diseases, Ninth Revision, Clinical Modification (ICD-9-CM). [13] Female RNs were followed until the development of cancer, death, dropping out from the NHI program, or the end of the year 2010.

The risk of cancer in the female RN cohort was determined using a standardized incidence ratio (SIR), which is defined as the observed number of cancer occurrences divided by the expected number. The expected number of cancers was calculated by multiplying the national incidence rate of cancers, stratified by gender, calendar year, and age in 5-year intervals, by the corresponding stratum-specific person-time accrued in the cohort. The incidence rates of cancers among the general population were obtained from the Taiwan Cancer Registry. The 95% confidence intervals (CIs) for the SIRs were estimated under the assumption that the number of cancers followed a Poisson probability distribution. We determined the SIRs for subgroups based on age. SIRs were also estimated for each cancer type. Finally, in order to compare the use of Pap smears by both the RNs and the general population, we selected female RNs in the LHID 2000 from January 1, 2000 to December 31, 2010 and recruited an age-matched control group from the LHID 2000 at a ratio of 1∶4. The average number of Pap smears in the two cohorts was compared using the paired t test.

Data extraction and computations were performed using the Perl programming language (version 5.12.2). Microsoft SQL Server 2005 (Microsoft Corp., Redmond, WA, USA) was used for data linkage, processing, and sampling. All statistical analyses were performed using SPSS statistical software version 17.0 for Windows (SPSS, Inc., Chicago, Illinois). Statistical significance was defined as a *p* value of less than 0.05.

## Results

### Patient Demographics

Overall, the cohort was observed for 1,371,910 person-years from 2000–2010. A total of 184,809 female RNs were identified. The median age at enrollment was 25.05 years (interquartile range, 22.32–32.14 years), and the median follow-up period was 7.86 years (interquartile range, 4.64–11.00 years). Nurse demographics are shown in Table 1.

**Table 1:** Characteristics of female registered nurses.

Characteristics	
No. of patients	184,809
Person-years at risk	1,371,910
Median follow-up, years (interquartile range)	7.86 (4.64–11.00)
Median age, years (interquartile range)	25.05 (22.32–32.14)
Distribution per age group, years	
20–39	165,435 (89.52%)
40–59	18,829 (10.19%)
≥60	545 (0.29%)

### All Cancers

A total of 2,077 cancers occurred within the observation period. Compared with the general population, female RNs had a higher overall cancer risk, with an SIR of 1.10 (95% CI 1.05–1.15). A subgroup analysis by age showed that an increased SIR was observed for nurses aged between 40–59 years (1.14, 95% CI 1.08–1.21). The results of the subgroup analysis are summarized in Table 2.

**Table 2:** Standardized incidence ratios (SIRs) according to age of female registered nurses.

	Number of cases of cancer		
Characteristics	Observed	Expected	SIR (95% CI)
All cancers	2077	1885.11	1.10 (1.05–1.15)
Age group, years			
20–39	861	799.72	1.08 (1.00–1.15)
40–59	1123	983.67	1.14 (1.08–1.21)
≥60	93	101.73	0.91 (0.74–1.12)

SIR Standardized incidence ratio; CI confidence interval.

### Specific Cancer Types

The cancer types most commonly observed in the female registered nurse cohort were breast (n = 827), followed by thyroid (n = 239), then colon and rectum (n = 174). An increased SIR was observed for breast (1.28, 95% CI 1.19–1.37), thyroid (1.26, 95% CI 1.10–1.43), lung and mediastinum (1.36, 95% CI 1.13–1.62), and uterine cancers (1.23, 95% CI 1.01–1.49) in the nurses. However, a decreased SIR was found for cervix (0.48, 95% CI 0.37–0.61) and liver and biliary tract cancers (0.68, 95% CI 0.50–0.90). SIRs for specific types of cancers are presented in Table 3.

**Table 3:** Standardized incidence ratios (SIRs) for specific cancer types among female registered nurses.

	Number of cases of cancer		
Site of cancers	Observed	Expected	SIR (95% CI)
All cancers	2077	1885.11	1.10 (1.05–1.15)
Head and neck	78	77.57	1.01 (0.79–1.25)
Digestive	294	291.97	1.01 (0.90–1.13)
Esophagus	2	2.95	0.68 (0.08–2.45)
Stomach	52	49.22	1.06 (0.79–1.39)
Colon and rectum	174	153.23	1.14 (0.97–1.32)
Anus	1	1.38	0.73 (0.02–4.05)
Liver and biliary tract	49	72.11	0.68 (0.50–0.90)
Pancreas	16	13.08	1.22 (0.70–1.99)
Lung and mediastinum	124	91.33	1.36 (1.13–1.62)
Bone and Soft tissue	21	25.83	0.81 (0.50–1.24)
Skin	30	22.60	1.33 (0.90–1.89)
Breast	827	647.14	1.28 (1.19–1.37)
Genitourinary	299	373.48	0.80 (0.71–0.90)
Cervix	70	145.55	0.48 (0.37–0.61)
Uterus	107	86.72	1.23 (1.01–1.49)
Ovary	96	104.02	0.92 (0.75–1.13)
Bladder	10	14.15	0.71 (0.34–1.30)
Kidney	16	23.04	0.69 (0.40–1.13)
Thyroid	239	189.80	1.26 (1.10–1.43)
Hematologic malignancies	102	95.95	1.06 (0.87–1.29)
All Others	63	69.42	0.91 (0.70–1.16)

SIR Standardized incidence ratio; CI confidence interval.

### Use of the Pap Smear by RNs and the General Population

In all, 7,778 female RNs and 31,112 age- and sex-matched controls were selected from the LHID 2000. During the follow-up period, the Pap smear was used 5.80 times per person among the female RNs aged 35 years or older and 5.50 times per person among the controls aged 35 years or older (paired t test, *p* = 0.009).

## Discussion

The study included 184,809 female RNs with a follow-up of 1,371,910 person-years. The data showed a higher overall cancer risk in female nurses relative to the general population, especially nurses aged between 40–59 years. The risks of breast, thyroid, lung and mediastinum, and uterine cancers specifically were higher in female RNs. However, the risks of cervix and liver and biliary tract cancers were lower. In addition, RNs aged 35 years or older used Pap smears more frequently than the general population.

The current study design included an unbiased subject selection and SIR estimations with matching for age, gender, and calendar year. Because participation in the NHI is mandatory and all residents of Taiwan can access healthcare with low co-payments, referral bias is essentially removed and follow-up is complete. To apply for a cancer catastrophic illness certificate, pathologic proof of malignancy is mandatory, and laboratory and imaging studies should also be provided. Patients with a certificate of catastrophic illness can be exempted from related medical expenses, especially hospital costs. Therefore, the cancer diagnoses in this study were not only reliable, but also exhaustive.

To the best of our knowledge, this is the largest population-based study of Taiwanese to evaluate the risk of cancer among female nurses. Although some large retrospective studies have addressed the association between nurses and cancer risk, their results were inconsistent. [4]–[10], [14]–[21] A study of 92,140 RNs in Denmark showed higher risks for breast, brain and nervous system cancer, melanoma, and other skin cancers, but no increase in overall cancer risk. Furthermore, significantly lower risks were observed for alcohol- and tobacco-related cancers. [17] In addition, a study of 58,125 nurses in British Columbia showed that a higher risk of malignant melanoma only was found in RNs. [14] A study of 68,336 nurses in the United States reported nurses working 10 years or more on rotating night shifts were associated with a 14% decreased risk of skin cancer compared with nurses never working night shifts. [9] In this US study, a lower risk of skin cancer was noted only in darker-haired nurses, when the nurses were stratified by hair color. The finding of this study suggests that both genetic and environmental factors may have an impact on cancer risk in nurses. Different study designs may partly explain these controversial results, but genetic differences between racial or ethnic groups should be taken into consideration. [22]–[25] Therefore, we conducted a nationwide population-based retrospective study to evaluate the risk of cancer among Taiwanese female RNs.

The current study found that overall cancer risk among RNs was higher than in the general population. The nature of the work performed by nurses makes shift and night work inevitable. Exposure to light at night and a disruption of the circadian rhythm have been hypothesized to increase the risk of cancer. [4]–[10] In 2007, the International Agency for Research on Cancer classified shift work involving disruption of circadian rhythms as a probable human carcinogen, 2A. [26] Besides, being a nurse implies the possibility of exposure to different carcinogens such as antineoplastic drugs, sterilizing gases, ionizing radiation, and viruses associated with various cancers. [27]–[30] These types of exposure might contribute differently to specific stages of cancer development and to certain cancer types, and increase cancer risk.

In the present study, we found that the risk of breast cancer among female RNs was higher than in the general population; this result was similar to those of many previous studies. [6], [7], [10], [17], [18], [27] The contributing cause being proposed mostly involves the night-time light exposure and decreases in melatonin, a circadian rhythm hormone. It is hypothesized that melatonin influences patterns of sex hormone production, such as increasing circulating estrogens, that in turn increase breast cancer risk. [31] In addition, exposure to ionizing and non-ionizing radiation has been hypothesized to increase the risk of breast cancer, [32], [33] and it has been estimated that about 10% of RNs are potentially exposed to ionizing radiation in their work. [34] A higher risk of thyroid cancer was also found in our study. Since the thyroid gland is very sensitive to radiation [35] and an excess of thyroid cancer had been reported among Chinese radiography workers, [36] it is reasonable to assume that the higher risk of thyroid cancer in our study cohort was partially due to exposure to radiation.

In our study, the risk of lung cancer was higher in female RNs, contrary to the results of many Western studies that revealed a lower risk of tobacco-related cancers, including lung cancer. [17], [18], [37] These studies attributed the lower risk of lung cancer to the lower prevalence of smoking among nurses than in the general population. However, given that the prevalence of cigarette smoking in Taiwanese women is very low, compared with Caucasian women (3–4% vs. approximately 28%, respectively), [38] the difference in the prevalence of smoking between the nurses and the general population might be minimal in the Taiwanese group. So, we did not find that the risks of other smoking-related malignancies, such as head and neck cancer, esophageal cancer and urinary system cancer, were lower as in these studies, and the risk of lung cancer was higher in our work possibly due to shift work and potential exposure to carcinogens.

We found that the risk of cervical cancer was lower in female RNs. The use of the Pap smear could explain this finding in part. The Pap smear for cervical cancer has been credited with dramatically reducing the incidence and mortality of cervical cancer in developed countries. [39] The NHI program in Taiwan provides free annual cervical cancer screening to women aged 30 years and older. We found that RNs aged 35 years or older used the Pap smear more frequently than the general population, and their risk of cervical cancer was thereby potentially lower.

A higher risk of uterine cancer (SIR 1.23, 95% CI 1.01–1.49, *p* = 0.04) and a lower risk of liver and biliary tract cancer (SIR 0.68, 95% CI 0.50–0.90, *p* = 0.005) were noted in our work. However, when taking into account multiple comparisons, the *p*-values of the above results did not have statistical significance (a *p*-value equal to or less than 0.05 divided by 22, approximately 0.002, was considered significant when adjusting with the Bonferroni correction) and the values may be chance findings.

The current study also found an increased SIR in female RNs aged 40–59 years. This is in agreement with the findings of two studies in which higher breast cancer risk was limited to postmenopausal nurses. [8], [40] A higher genetic component in premenopausal breast cancer combined with a shorter working history and a shorter latency period among younger nurses, are reasons for assuming that occupational exposures are more relevant for postmenopausal breast cancer. Furthermore, we did not find that cancer risk was higher in female RNs aged over 60 years. This might be due to the smaller number of RNs (n = 552) in this age group, which decreased statistical power for the group.

There are several limitations to this large population-based study. First, many demographic variables were not available, such as socioeconomic status, family history of cancer, reproductive patterns including early age at first pregnancy and multiparity, diet, cigarette smoking, and alcohol use. Second, actual occupational information, such as nursing specialty, workplace, cumulative number of lifetime night shifts, lifetime average number of night shifts per month, and so on, could not be obtained. All the above factors have been linked with cancer risks in nurses [7], [16].

In conclusion, this population-based retrospective cohort study found a higher cancer risk among female RNs compared with the general population. The risks of breast, lung, thyroid and uterine cancers specifically were higher and the risks of cervix and liver cancers were lower among female RNs. Further large, unbiased population-based prospective studies are needed to investigate the association between nurses and cancer risk.
